# Influence of Extrusion Screw Speed and CNT Concentration on the Mechanical and EMI Properties of PC/ABS Based Nanocomposites

**DOI:** 10.3390/ma17112625

**Published:** 2024-05-29

**Authors:** Caolan Jameson, Declan M. Devine, Gavin Keane, Noel M. Gately

**Affiliations:** 1 PRISM Research Institute, Technological University of the Shannon: Midlands Midwest, Athlone Campus, University Road, N37 HD68 Athlone, Ireland; 2 Centre for Industrial Service & Design, Technological University of the Shannon: Midlands Midwest, Athlone Campus, University Road, N37 HD68 Athlone, Ireland; 3 Applied Polymer Technologies Gateway, Technological University of the Shannon: Midlands Midwest, Athlone Campus, University Road, N37 HD68 Athlone, Ireland

**Keywords:** injection moulding, polycarbonate/acrylonitrile-butadiene-styrene (PC/ABS), design of experiments (DoE), mechanical properties, extrusion screw speed, electromagnetic interference shielding effectiveness (EMI SE)

## Abstract

This study investigates the effect of extrusion screw speed and carbon nanotube (CNT) concentration on the thermal, mechanical, and electromagnetic interference shielding effectiveness (EMI SE) properties of Polycarbonate (PC)/acrylonitrile-butadiene-styrene (ABS) and its polymer nanocomposites (PNCs) by means of design of experiments (DoE) approach. A masterbatch method was employed to obtain the best dispersion of the CNTs throughout the polymer matrix. This study evaluates the thermo-mechanical characterisation of the polymers and PNCs at varying screw speeds to assess filler matrix bonding. The results highlight that CNT concentration has a significant effect on all mechanical properties, while screw speed only affects the Charpy impact strength and flexural properties of the samples. Compounding at 200 rpm has the best flexural and tensile strength, which is attributed to the best filler matrix bonding (highest storage modulus) of the PNCs. The best EMI SE results were obtained at 10 wt.% CNTs. This research contributes valuable insights into the effect of CNT concentration and extrusion screw speed on the mechanical, thermal and EMI SE properties of PC/ABS and its PNCs.

## 1. Introduction

Polymers have seen great interest in the automotive industry due to their lightweight, versatility, and ease of processing. However, as EMI shielding materials, most polymers are intrinsically electrically insulative and need conductive fillers to part a conductive network throughout the polymer [1,2]. A blend of PC and ABS is commonly used in the electrical, telecommunications, and automotive industries due to the high thermal stability, toughness, processability, and good mechanical properties.

For polymer nanocomposites (PNCs), several factors can affect EMI SE. Achieving good dispersion of CNTs throughout the polymer matrix is crucial to obtaining the desired mechanical, electrical and EMI properties as CNTs tend to agglomerate together, which can negatively impact these properties [3]. Agglomerates generally undergo rupture or erosion mechanisms, which cause the nanofillers to disperse [4]. The production of uniformly dispersed nanocomposites has been achieved through the high shear and temperature conditions used during extrusion. Sriseubsai et al. [5] studied the effect of carbon black powder (CBp) and carbon black masterbatch (CBm) as conductive fillers in a PC/ABS matrix for EMI shielding applications. The authors found that when using PC/ABS as a shielding material for car audio components, the highest amount of CB (Carbon Black) is recommended, and PC should be the dominant polymer to attain the highest EMI SE. However, this research does not take mechanical properties into account, with a higher volume of CB filler resulting in lower impact properties. Of the conductive carbonaceous fillers, CNTs have achieved the highest electrical conductivity at the same weight fraction due to their lower percolation threshold [6]. This lower percolation threshold is due to their higher aspect ratio and high specific surface area [7]. Dal Lago et al. [6] found that CB and CNTs show preferential dispersion in the PC phase of PC/ABS. This preferential dispersion would allow for increased electrical conductivity in the nanocomposite phase, thus increasing EMI SE [8]. Han et al. [9] found preferential dispersion of the CNTs in the ABS phase due to the lower interfacial tension of the ABS/MWCNT composite compared to the PC/MWCNT composite. With the introduction of SAN-g-MAH into the PC/ABS blend, the ABS phase was more dispersed, and the EMI SE increased. The preferential dispersion to the ABS phase is present when the SAN-g-MAH is added to the composite. The Maleic anhydride contains a polar carbonyl group, which can interact with the polar ester group in PC [10]. This polarity of the carbonyl group could also have caused the preferential dispersion of the CNTs to the ABS phase. While Han et al. [9] found preferential dispersion of the CNTs in the ABS phase due to lower interfacial tension, Rostami et al. [11] found preferential dispersion in the PC phase while the ABS phase had lower interfacial tension. The authors found that the CNTs were localised at the interface of the PC and ABS phases. This localisation of CNTs around the PC phase caused a decrease in the damping factor due to the restriction of the PC chains [11].

Studies into the reduction of these CNT agglomerations using modification [12], compatibilisers [9,13], and optimisation of processing conditions [14,15] have been undertaken. A masterbatch approach results in better dispersion of the CNTs as the agglomerates are subjected to shear stresses twice [16]. The screw speed in extrusion is a critical factor that affects the dispersion of CNTs throughout a polymer matrix, through affecting the level of shear and compressive forces acting on the PNC. The optimal screw speed for a particular nanocomposite depends on the polymer matrix, CNT content and the desired properties of the nanocomposite. Kasaliwal et al. [4] studied the effect of screw speed and mixing time on the agglomerate dispersion in PC/Multiwalled-CNT (MWCNT) PNCs and found that agglomerate dispersion is better in composites prepared at higher screw speeds (300 rpm). Having a higher mixing time (40 min) also aided in the dispersion of MWCNTs with the best dispersion occurring in samples at screw speed 300 rpm and a mixing time of 40 min. While a high screw speed and high mixing times improve the dispersion of the MWCNTs, the effect of shear degradation on the mechanical properties was not studied. Jyoti et al. [17] also studied the effect of increasing MWCNT content on the electrical conductivity, EMI SE, and tensile properties of ABS/MWCNTs varying from 1 wt.% to 10 wt.%. The highest EMI SE (36 dB) and tensile modulus (39% increase from pure ABS) was obtained at 10 wt.% MWCNT. However, a drop in tensile strength in the 10 wt.% MWCNT samples were theorised by the authors to be caused by MWCNT agglomerations in the ABS matrix. The agglomerations observed in this study may be caused by an unoptimised extruding process. It can be inferred that a mixing time of 3 min and a screw speed of 150 rpm, as indicated from Kasaliwal et al. [4], would likely have resulted in large agglomerations to remain in the matrix. These agglomerations would have created defects in the polymer matrix, which would lower the mechanical properties of the PNC [3]. Babal et al. [3] assessed the EMI SE, flexural properties, and tensile properties of PC reinforced with MWCNTs varying from 0.5 wt.% to 10 wt.%. This work achieved results equivalent to those of Jyoti et al. [17], with a reduction in tensile strength occurring from 2 wt.% to 5 and 10 wt.% MWCNT PNCs. This trend also occurred in the flexural strength of the PNCs. The authors also attributed this reduction in properties to the formation of agglomerations of MWCNTs in the PC matrix. The mixing time for this study was also 3 min and a screw speed of 100 rpm was used. This screw speed did not achieve adequate dispersion of CNTs in a 9-min mixing time [4], so large agglomerations may be present in Babal et al. [3] 5 and 10 wt.% MWCNT/PC PNCs. Further work into the improvement of dispersion of CNTs into a polymer matrix is warranted as higher screw speeds improve dispersion of CNTs but the effect of the shear stress associated with this process has not been studied.

The present work evaluates the mechanical, thermal and EMI properties of the nanocomposites with three mass fractions of 0, 5 and 10 wt.% of the CNT blended into a PC/ABS matrix using a twin-screw extruder with three different screw speeds 100, 200, and 300 rpm based on [4,15].

## 2. Materials and Methods

### 2.1. Materials

A blend of Polycarbonate (PC) and acrylonitrile-butadiene-styrene (ABS) known as Bayblend FR3010 was supplied in pellet form by Covestro (Covestro AG, Leverkusen, Germany). This material has a higher proportion (>50 wt.%) of PC relative to ABS. The density of the PC/ABS is 1.18 g/cm^3^ and a melting temperature range of 240–270 °C. MWCNTs were obtained by Nanocyl (Nanocyl S.A, Sambreville, Belgium), under the trade name NanocylTM NC7000. The reported characteristics of the MWCNTs are as follows: Average nanotube length of 1.5 μm; average nanotube diameter of 9.5 nm, Volume resistivity of 10^−4^ Ωcm; carbon purity > 90%; surface area of 250–300 m^2^/g and transition metal oxide of <1%. Both PC/ABS and the MWCNT filler were dried at 80 °C for 4 h.

### 2.2. Design of Experiments (DoE)

For the Design of Experiments (DoE), the selected parameters and their values are given in Table 1. For the evaluation of the DoE, the software Minitab version 20.4 was used. A full factorial DoE using the run order is shown in Table 2, with 5 replicates for each parameter. For tensile, impact, and flexural testing, samples were manufactured to ASTM D638-14 type I [18], D6110-18 [19] and D790-17 [20] specifications, respectively. For dynamic Mechanical thermal analysis (DMA), rectangular samples measuring 70 mm × 12 mm × 2 mm were manufactured. Samples for EMI SE analysis were manufactured using a mould with a square plaque of 100 mm width and 2 mm thickness.

### 2.3. Material Processing

#### 2.3.1. Extrusion

A 25 wt.% MWCNT PC/ABS masterbatch was prepared using the Brabender internal mixer (Brabender GmbH & Co. KG, Duisburg, Germany). The temperature was set at 270 °C and the screw speed was set to 50 rpm. The different weight fractions of 100:0, 80:20, and 60:40 for PC/ABS: MWCNT masterbatch were prepared for extrusion to achieve 0, 5, and 10 wt.% MWCNT. Extrusion was performed using am EUR. EX. MA mini-E lab 22 high-temperature compounder (Eurotech Extrusion Machinery, Tradate VA, Italy) with 21.7 mm diameter screws and a length-diameter (L/D) ratio of 40/1. The temperature profile was set at (from die to feeder) 265/260/250/245/240/240/220/160 °C and remained constant for all batches of materials. Three different screw speeds were selected for this study: 100, 200, and 300 rpm. The feed rate of the MWCNT masterbatch was set using the Movacolor MCTwin gravimetric feeder (Movacolor, Sneek, The Netherlands). Extruded strands of the PC/ABS and the composite were drawn through a water bath and fed into a pelletiser.

#### 2.3.2. Injection Moulding

The pellets were dried at 80 °C for 4 h before injection moulding. The injection moulding was performed using the Arburg Allrounder injection moulding machine (Arburg GmbH & Co. KG, Lossburg, Germany). The temperature profile was set at (from die to feeder) 265/260/240/240/230/50 °C. The following moulding parameters were chosen and remained constant for all the materials used: Mould temperature of 75 °C; back pressure of 40 bar; injection pressure of 1500 bar; injection speed of 40 mm/s; holding pressure of 1100 bar; holding time of 5 s; cooling time of 30 s.

### 2.4. Archimedes’ Buoyancy Method

The density of the manufactured samples was determined using ASTM D792 standards [21], *n* = 5. Density calculations were performed at 20 °C. Theoretical densities of the pure PC/ABS and the nanocomposites samples were predicted using the rule of mixtures Equation (1).

(1) $$\rho_{C}=\rho_{m}\;V_{m}+\rho_{f}\;V_{f}$$

where the subscripts *c*, *f*, and *m* represent composite, fibre (carbon nanotubes), and matrix, respectively. The density of the CNTs used in this equation was obtained from Zhao et al. [22] which was 1.75 g/cm^3^.

### 2.5. Dynamic Mechanical Thermal Analysis (DMA)

DMA was used to measure storage modulus, loss modulus, and loss factor (tan delta) and were determined using TA Instrument Q800 DMA (TA Instruments, Eschborn, Germany) and a dual cantilever apparatus, *n* = 5. The analysis was conducted at a frequency of 1 Hz. The temperature range was ambient temperature to 150 °C at a heating rate of 3 °C/min.

### 2.6. Charpy Impact Testing

The impact tests were conducted using the Instron Ceast Resil 5.5 impact tester (Illinois Tool Works Inc., Glenview, IL, USA). The impact properties were determined using the ASTM D6110-18 standards. A 4-joule hammer was used. A minimum of five samples were tested for the pure PC/ABS and the nanocomposites at different screw speeds, as per the standard.

### 2.7. Tensile Testing

The tensile tests were conducted on both PC/ABS and nanocomposites for the three screw speeds. The samples were tested using the Zwick/Roell Z010 with a load cell of 10 kN, and the tests were carried out at room temperature (ZwickRoell Ltd., Ulm, Germany). The tensile properties were determined using the ASTM D638-14 standards. The test speed was set to 5 mm/min and a gauge length of 50 mm. A minimum of ten samples were tested for the pure PC/ABS and the nanocomposites at different screw speeds, as per the standard. A video extensometer was used to measure the modulus of the materials.

### 2.8. Flexural Testing

The flexural tests were conducted using the Zwick/Roell Z010 (ZwickRoell Ltd., Ulm, Germany). The flexural properties were determined using the ASTM D790-17 standards. A span-to-depth ratio of 16:1 and a test speed of 2.7 mm/min. A minimum of five samples were tested for the pure PC/ABS and the nanocomposites at different screw speeds, as per the standard.

### 2.9. Electromagnetic Interference Shielding Effectiveness (EMI SE)

The EMI SE of both PC/ABS and nanocomposites for the three screw speeds were evaluated in the frequency range between 1 and 3 GHz using a Rohde and Schwarz FSH6 spectrum analyser (Rohde & Schwarz GmbH & Co. KG, Munich, Germany). The samples were placed inside an anechoic chamber. The experimental arrangement consisted of positioning an antenna 3 m away from the emitter inside the anechoic chamber. The acrylic sample holder was positioned in front of the emitter so that the middle of the sample lined up with the direction of the EM wave and secured into place. A calibration of the spectrum analyser was conducted to eliminate noise. The attenuation was recorded for with and without the sample holder as a control. Samples were placed inside the sample holder, and the anechoic chamber was sealed. The attenuation was allowed to settle for 1 min, and the attenuation across the frequency was recorded. A minimum of 5 samples were tested for the pure PC/ABS and the nanocomposites at different screw speeds.

### 2.10. Differential Scanning Calorimetry (DSC)

A NETZSCH DSC 214 Polyma (Erich NETZSCH B.V. & Co. Holding KG, Selb, Germany) was used to detect the glass transition temperature (Tg) of PC/ABS and its PNCs. The samples were tested in duplicate at a heating rate of 10 °C/min, from ambient to 300 °C. The samples were held at 300 °C for 10 min to remove thermal history and cooled to ambient at a rate of 10 °C/min. A second heating cycle was also assessed at a rate of 5 °C/min to 300 °C. Aluminium standard NETZCH pans and lids and a sample weight of 5–10 mg were used.

### 2.11. X-ray Diffraction (XRD)

X-ray Diffraction (XRD) was used to identify the crystalline phases present in the material and the ways in which the addition of CNTs to the PC/ABS affected their crystallinity. Testing was conducted in triplicate with a Malvern Panalytical Aeris Diffractometer (Malvern Panalytical Ltd., Almelo, The Netherlands) from 4 to 70° with a step size of 0.043° 2θ and a scan speed of 0.055 ° s^−1^.

## 3. Results

### 3.1. Density Measurements

**Figure 1:**
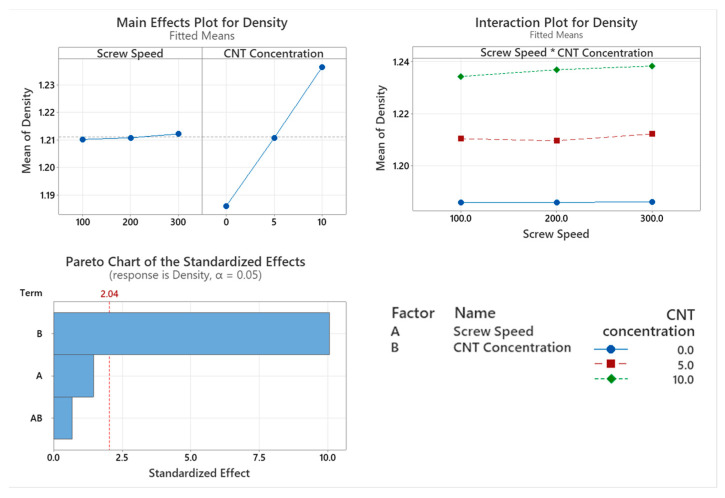
Statistical analysis of density data: Main effect plot, interaction plot and Pareto chart. Based on the data, CNT concentration caused a statistically significant increase in density.

The rule of two mixtures was used to measure the theoretical density of the material. While the baseline density for the virgin polymer was 1.18 g/cm^3^, the measured density of this material was 1.186 g/cm^3^. This value was used to calculate the theoretical density of the nanocomposites, which were 1.2142 g/cm^3^ and 1.2424 g/cm^3^ for 5% CNT and 10% CNT PNCs, respectively. As seen in the Pareto chart in Figure 1, CNT concentration significantly affects the density of the polymer (*p* < 0.001), while screw speed (*p* = 0.155) and the interaction between the two factors (*p* = 0.499) did not significantly affect the density of the PNCs. From the main effect plot in Figure 1, there is a direct linear relationship between CNT concentration and PNC’s density.

### 3.2. Dynamic Mechanical Thermal Analysis (DMA)

#### 3.2.1. Storage Modulus

**Figure 2:**
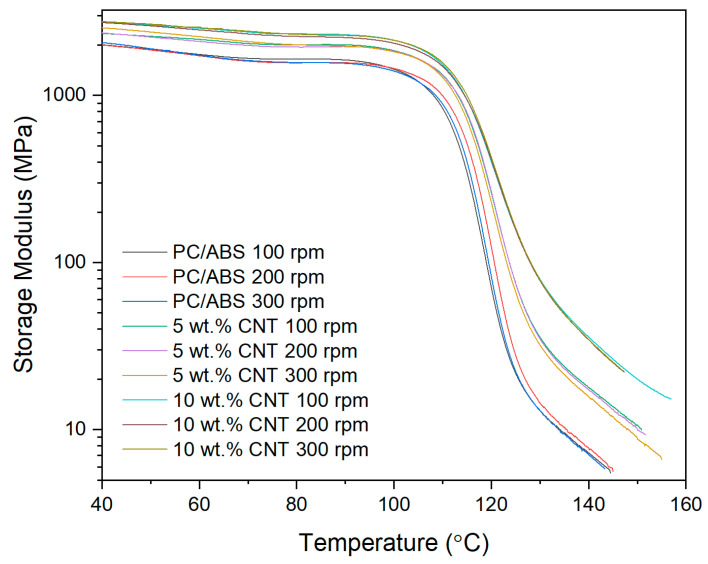
Storage modulus thermograms of PC/ABS and its PNCs at varying screw speeds obtained using DMA.

**Figure 3:**
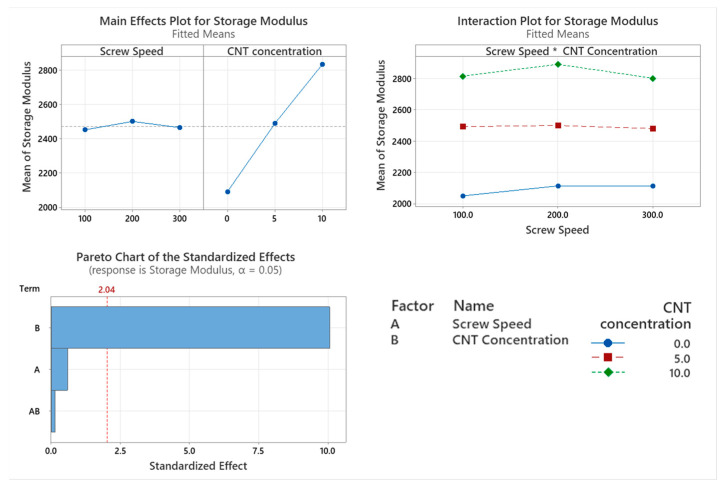
Statistical analysis of Storage Modulus DMA data: Main effect plot, interaction plot and Pareto Chart for Storage Modulus. Based on the data, CNT concentration had a statistical increase in results.

The storage modulus of the PNCs with varying screw speed were assessed using DMA as shown in Figure 2. The only factor affecting the storage modulus is the CNT concentration (*p* < 0.001), which can be seen in the Pareto chart in Figure 3. Screw speed (*p* = 0.56) and the interaction between the two factors (*p* = 0.889) did not significantly affect the storage modulus of the PNC. From the main effect plot in Figure 3, there is a direct linear relationship between storage modulus and CNT concentration.

#### 3.2.2. Loss Modulus

**Figure 4:**
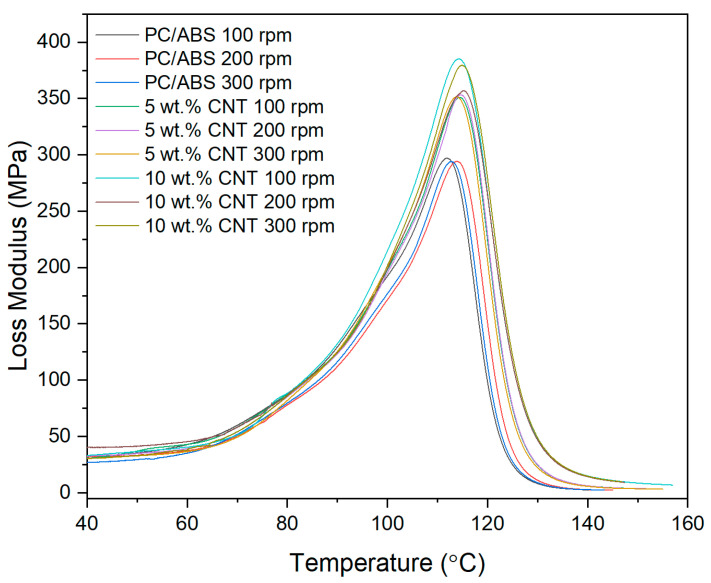
Loss modulus thermograms of PC/ABS and its PNCs at varying screw speeds obtained using DMA.

**Figure 5:**
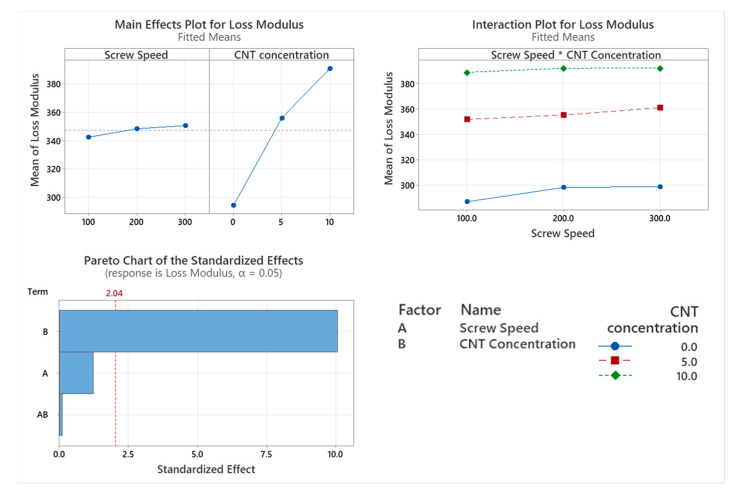
Statistical analysis of Loss Modulus DMA data: Main effect plot, interaction plot and Pareto Chart for Loss Modulus. Based on the data, CNT concentration had a statistical increase in results.

The viscous response (Loss Modulus) of the PNCs with varying screw speed were assessed using DMA, as shown in Figure 4. The only factor affecting the storage modulus is the CNT concentration (*p* < 0.001). Screw speed (*p* = 0.231) and the interaction between the two factors (*p* = 0.913) did not significantly affect the storage modulus of the PNC. From the main effect plot in Figure 5, there is a direct linear relationship between storage modulus and CNT concentration.

#### 3.2.3. Tan δ

**Figure 6:**
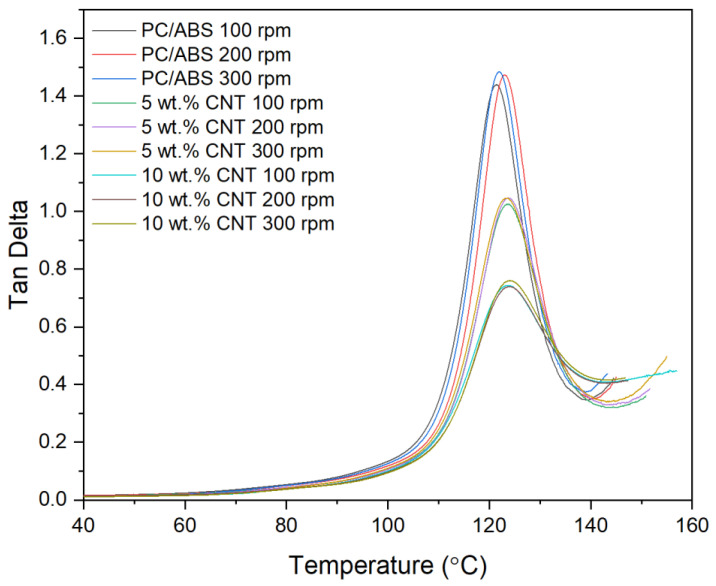
Tan δ thermograms of PC/ABS and its PNCs at varying screw speeds obtained using DMA.

**Figure 7:**
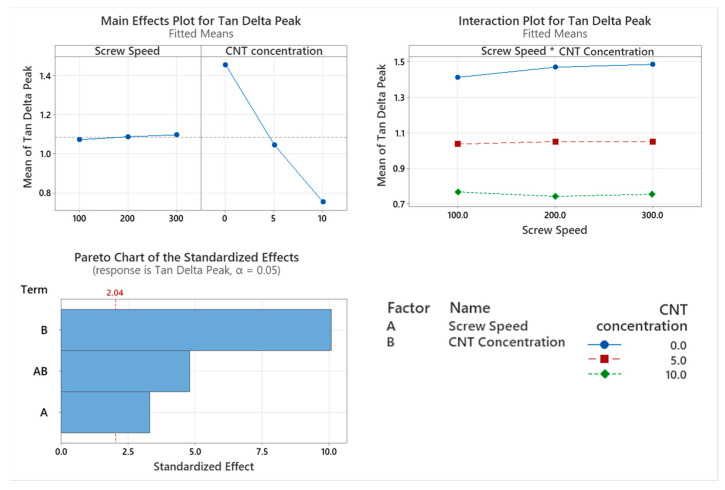
Statistical analysis of Tan δ DMA data Main effect plot, interaction plot and Pareto Chart for Tan Delta Peak. Based on the data, all factors had a significant influence on results, with CNT causing the highest reduction in the Tan delta peak.

The damping factor or tan δ as a function of temperature of the PNCs with varying screw speed were assessed using DMA as shown in Figure 6. The CNT concentration of the PNC had a significant effect on the damping factor of the material (*p* < 0.001). Screw speed (*p* = 0.002) along with the interaction between the two factors (*p* < 0.001) also had a significant effect on the damping factor of the material. As seen in the main effects plot in Figure 7, there is an inversely linear relationship between the damping factor of the material and the CNT concentration of the PNC.

#### 3.2.4. Glass Transition Temperature (Tg)

**Figure 8:**
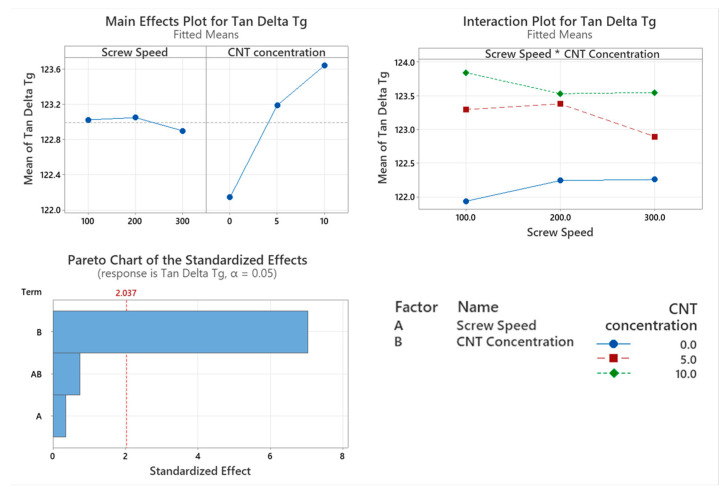
Statistical analysis of glass transition temperature DMA data: Main effect plot, interaction plot and Pareto Chart for glass transition temperature. Based on the data, CNT concentration had a statistical increase in the results.

The Tan delta peak temperature was chosen as the glass transition temperature (Tg) for this study. The Tg of the PNC was assessed with CNT concentration having a significant effect on the Tg (*p* < 0.001). Screw speed (*p* = 0.726) and the interaction between the two factors (*p* = 0.459) did not significantly affect the Tg of the PNCs. From the Main effect plot in Figure 8, there is a direct linear relationship between Tg and the CNT concentration of the PNC.

### 3.3. Charpy Impact Testing

**Figure 9:**
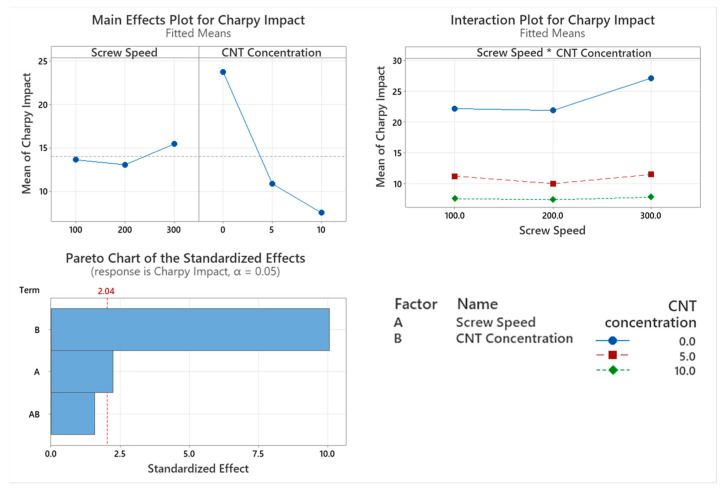
Statistical analysis of Charpy impact strength data Main effect plot, interaction plot and Pareto Chart for Charpy impact strength. Based on the data CNT concentration and screw speed had a significant influence on results, with CNT having the greatest influence on results.

The Charpy impact strength of the PNCs with varying screw speed were assessed with the main factor significantly affecting impact strength being CNT concentration (*p* < 0.001), which can be seen in the Pareto chart in Figure 9. Screw speed also significantly affects impact strength (*p* = 0.0032), but the interaction between these two factors does not significantly affect Charpy impact strength (*p* = 0.126). From the main effect plot in Figure 9, it is evident that the higher the CNT concentration in the PNC, the lower the Charpy impact strength. From the main effect plot we can also see that the higher screw speed achieves a higher impact strength.

### 3.4. Tensile Testing

**Figure 10:**
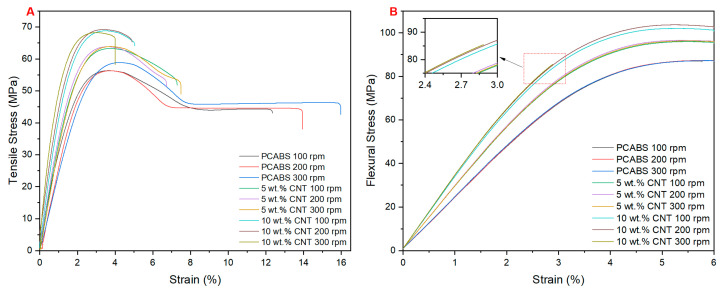
Tensile (**A**) and flexural (**B**) stress/strain graphs for PC/ABS and its PNC at varying screw speeds.

Figure 10A shows the stress/strain curve obtained through tensile testing. With the incorporation of CNTs into the PC/ABS matrix, the PNC saw a rise in tensile modulus, indicating enhanced stiffness, while the elongation at break reduced, indicating increased brittleness.

#### 3.4.1. Tensile Modulus

**Figure 11:**
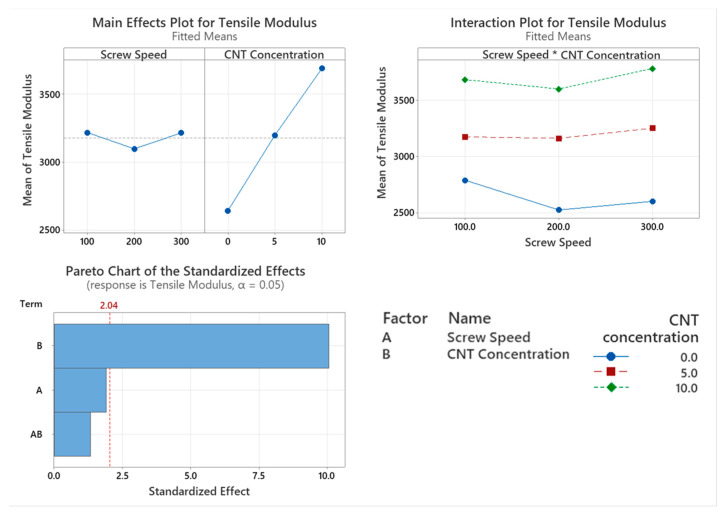
Statistical analysis of tensile modulus data Main effect plot, interaction plot and Pareto Chart for tensile modulus. Based on the data, CNT concentration caused a statistical increase in the results.

The tensile modulus of the PNCs with varying screw speed were assessed with the main factor significantly affecting tensile modulus being CNT concentration (*p* = 0.000), as shown in the Pareto chart in Figure 11. Screw speed has a moderate influence on tensile modulus. However, it is not a statistically significant factor (*p* = 0.064). The interaction between the two factors did not significantly affect the tensile modulus of the polymer (*p* = 0.192). From the main effect plot in Figure 3, there is a direct linear relationship with CNT concentration, with higher wt.% CNTs achieving a higher tensile modulus.

#### 3.4.2. Tensile Strength

**Figure 12:**
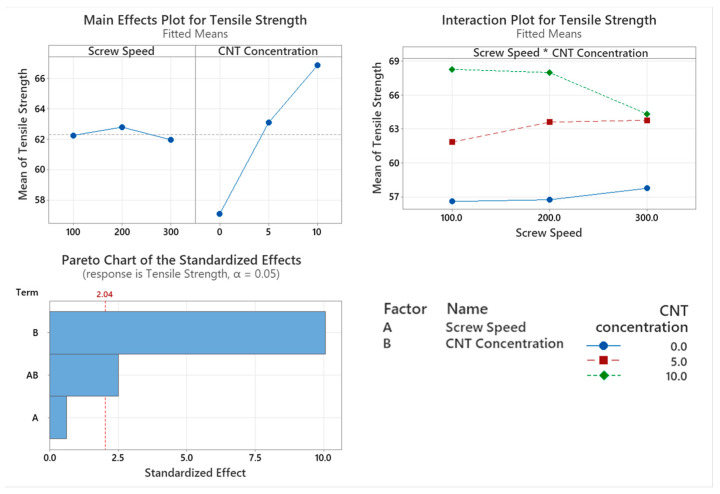
Statistical analysis of Tensile Strength data Main effect plot, interaction plot and Pareto Chart for Tan Delta Peak. Based on the data CNT concentration and the interaction of screw speed and CNT concentration had a significant influence on results, with CNT having the greatest influence on results.

The tensile strength of the PNCs with varying screw speed were assessed with the main factor significantly affecting tensile strength being CNT concentration (*p* < 0.001), as shown in Figure 12. Screw speed is not a statistically significant factor that affects the tensile modulus of the polymer (*p* = 0.535). The interaction between CNT concentration and screw speed did significantly affect the tensile strength (*p* = 0.017). From the main effect plot in Figure 12, there is a direct linear relationship with CNT concentration, with higher wt.% CNTs achieving a higher tensile strength. From the interaction plot in Figure 12, the effect of Screw speed and CNT concentration can be observed with a drop in tensile strength for higher both screw speeds and CNT concentration.

#### 3.4.3. Elongation at Break

**Figure 13:**
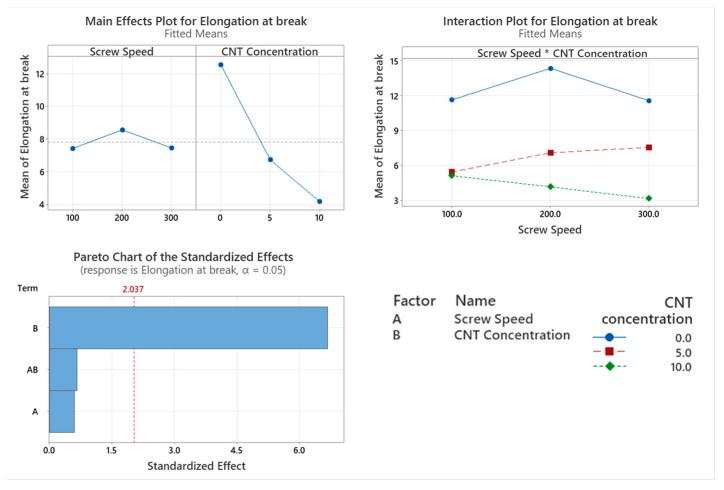
Statistical analysis of elongation at break data: Main effect plot, interaction plot and Pareto Chart. Based on the data, CNT concentration caused a statistically significant reduction in elongation at break.

The elongation at break of the PNCs with varying screw speed were assessed with the main factor significantly affecting elongation at break being CNT concentration (*p* < 0.001), as shown in the Pareto chart in Figure 13. Screw speed is not a statistically significant factor that affects the tensile modulus of the polymer (*p* = 0.550). The interaction between the two factors did not significantly affect the tensile modulus of the polymer (*p* = 0.514). There is an inversely linear relationship between CNT concentration and elongation at break.

### 3.5. Flexural Testing

Figure 10B shows the stress/strain curve obtained through flexural testing. With the incorporation of CNTs into the PC/ABS matrix, the PNC saw a rise in flexural modulus and strength, indicating enhanced stiffness. The 10 wt.% CNT samples compounded at 300 rpm saw a reduction in strain at break, indicating increased brittleness in this sample.

#### 3.5.1. Flexural Modulus

**Figure 14:**
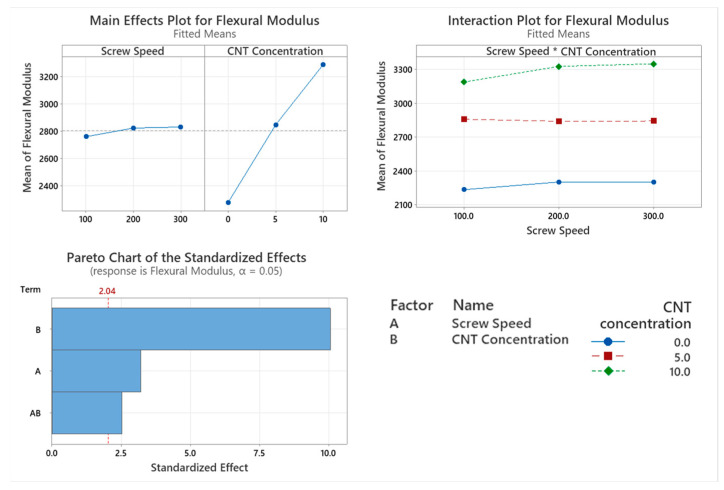
Statistical analysis of flexural modulus data Main effect plot, interaction plot and Pareto Chart for flexural modulus. Based on the data, all factors had a significant influence on results, with CNT causing the highest increase in flexural modulus.

The flexural modulus of the PNCs with varying screw speed were assessed with the main factor significantly affecting flexural modulus being CNT concentration (*p* < 0.001), as shown in the Pareto chart in Figure 14. Screw speed is a statistically significant factor that affects the flexural modulus of the polymer (*p* = 0.003). The interactions of the two factors significantly affect the flexural modulus of the polymer (*p* = 0.016). From the main effects plot in Figure 14, there is a direct linear relationship between CNT concentration and flexural modulus.

#### 3.5.2. Flexural Strength

**Figure 15:**
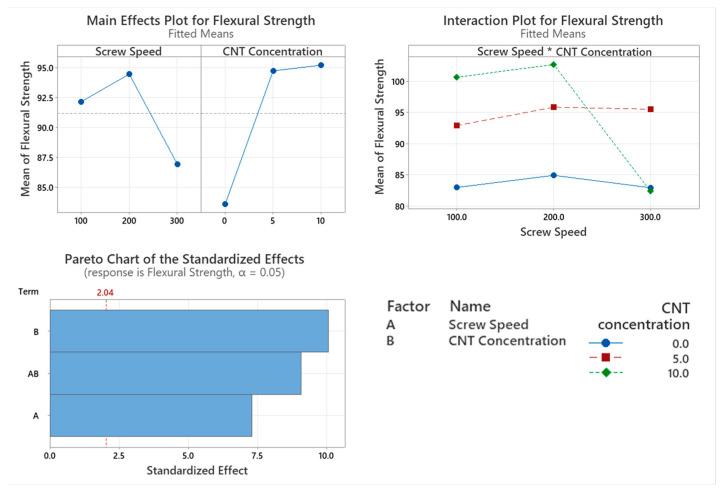
Statistical analysis of flexural strength data Main effect plot, interaction plot and Pareto Chart for flexural strength. Based on the data, all factors had a significant influence on results, with CNT causing the highest increase in flexural strength.

The flexural strength of the PNCs with varying screw speed were assessed with the main factor significantly affecting flexural strength being CNT concentration (*p* < 0.001), as shown in the Pareto chart in Figure 15. Screw speed was a significant factor that affects the flexural strength of the PNCs (*p* < 0.001). The interaction between the two factors significantly affects the flexural strength of the PNC (*p* < 0.001). There is a significant drop in flexural strength as the screw speed increases from 200 to 300 rpm. The flexural strength of the PNC increases as CNT content increases.

### 3.6. Electromagnetic Interference Shielding Effectiveness (EMI SE)

**Figure 16:**
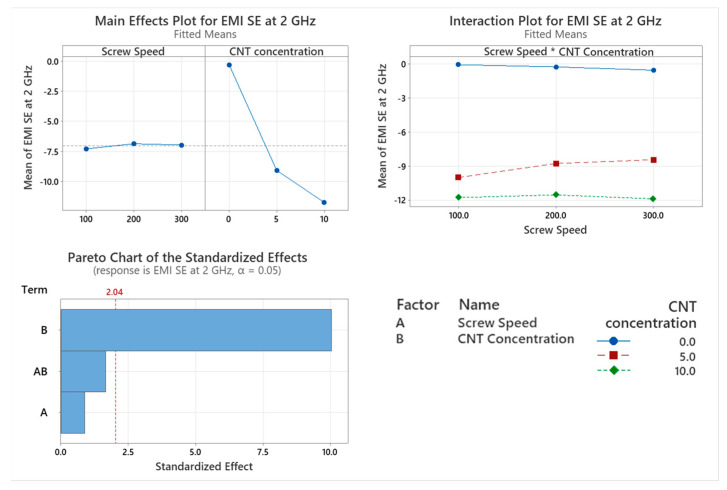
Statistical analysis of EMI SE at 2 GHz data: Main effect plot, interaction plot and Pareto Chart. Based on the data, CNT concentration caused a statistically significant reduction in EMI SE at 2 GHz.

The EMI SE of the PNCs at 2 GHz with varying screw speed were assessed with the main factor significantly affecting EMI SE being CNT concentration (*p* < 0.001), as shown in Figure 16. There is no significant effect on EMI SE caused by screw speed (*p* = 0.368) or the interaction between the two factors (*p* = 0.108). There is a direct linear relationship between EMI SE and CNT concentration, with 10 wt.% CNT achieving the highest attenuation.

### 3.7. Differential Scanning Calorimetry (DSC)

**Figure 17:**
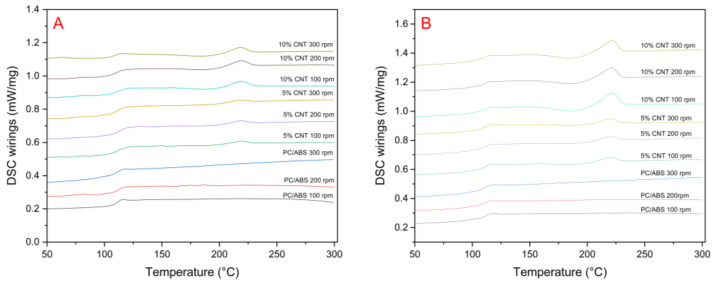
DSC thermographs of (**A**) first heating and (**B**) second heating of PC/ABS and PC/ABS PNC at different screw speeds.

The Midpoint of the glass transition temperature (Tg) of the samples were assessed using a DSC (Figure 17). The Tg of the samples is shown in Table 3. The highest Tg of the samples was obtained by the 5 wt.% CNT at 200 rpm screw speed (113.5 °C). The lowest Tg was obtained by the 10 wt.% CNT at 300 rpm screw speed (107.8 °C).

An endothermic peak is present in the PNC samples at approximately 218 °C, with the peak intensifying with increasing CNT concentration. In the second heating of the samples, a small exothermic peak is present in the 10 wt.% CNT samples, right before the endothermic peak at approximately 190 °C.

### 3.8. X-ray Diffraction (XRD)

**Figure 18:**
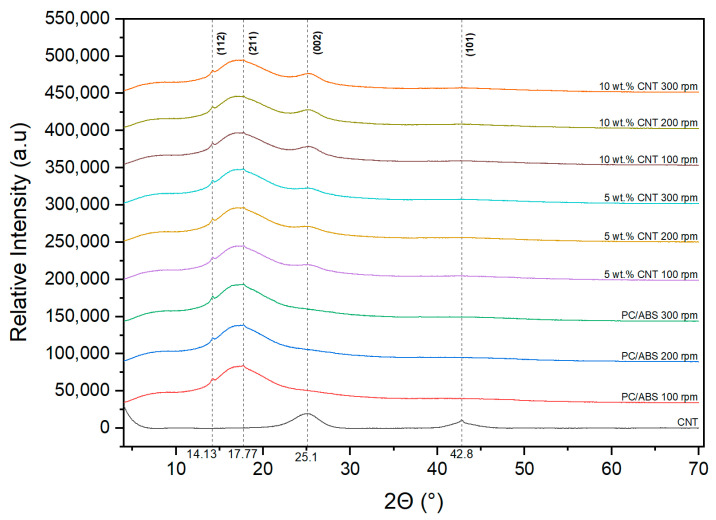
X-ray diffractograms of PC/ABS, CNTs and, PC/ABS PNCs at different screw speeds.

Figure 18 shows the XRD plots for CNT, PC/ABS and PCABS PNC samples. The peaks present at 14.13° and 17.77° in all the samples correspond to the 112 and 211 planes of a PC crystal. The peak present at 25.1° in the CNT plot (002 plane) corresponds to the crystallographic plane of the hexagonal carbon lattice within the CNTs. This peak suggests that the CNTs have a well-defined and ordered structure. The peak present at 42.8° (101 plane) corresponds to the periodic arrangement of carbon atoms along the circumference of the CNT. For the PNC samples, the 002 peak intensifies with increasing CNT concentration, while the 101 peak is not present.

## 4. Discussion

The experimental design utilised aimed to identify optimal concentrations of CNT and processing screw speed for the preparation of additively manufactured (AM) EMI shielding constructs. The study utilised a twin-screw extruder to disperse the CNT within the PC/ABS matrix at varying screw speeds. In doing so, two competing interactions were observed: (1) dispersion of the CNT within the matrix and (2) shear-generated changes to the polymer molecular chain. Interaction plots from various tests indicated that the CNT concentration had the most significant influence (either positive or negative) on the results observed.

Based on statistical modelling it was found that the introduction of CNTs into PC/ABS saw a significant increase in density, as would be expected since the density of CNT is 1.75 g/cm^3^ [22] compared to that of the polymer which was measured at 1.186 g/cm^3^. Screw speed did not significantly affect the density of the samples, as this result would not be influenced by changes in the polymer molecular chain.

The thermo-mechanical characterisation of PC/ABS and its PNC were assessed using DMA. The storage modulus (E’) is the measure of the energy stored elastically during deformation, while the loss modulus is the measure of energy converted to heat [23]. The introduction of CNTs into the PC/ABS matrix increased the PNCs’ ability to store energy elastically during deformation. The storage modulus of a PNC is highly affected by the composition and the interfacial adhesion between matrixes [24]. A higher initial modulus in a PNC can be attributed to good filler matrix bonding [25]. The highest storage modulus achieved was that of 10 wt.% CNT PNC compounded at 200 rpm, with a reduction in storage modulus observed at 300 rpm. This reduction is likely related to shear-induced reduction in polymer chain length. CNT concentration had a significant effect on the loss modulus of the PNC. Hameed et al. [26] observed similar peak broadening when using epoxy and glass fibre and reported that a higher loss modulus is due to the enhanced internal frictional interactions that enhance the dissipation of energy. These increased frictional interactions occurring throughout the PNC are due to the high aspect ratio and surface area of the CNTs [27]. The introduction of CNTs to the PC/ABS matrix has improved the viscoelastic properties of the PNC, with CNT concentration significantly affecting the Tan delta peak of the PNC. A lower Tan delta peak indicates a higher stored-to-loss energy ratio at the Tg. The reduction in the Tan delta peak (damping factor) with the increasing concentration of CNTs may relate to a strong hindrance in chain mobility caused by the entangled CNT networks [24].

Screw speed and the interaction between the two factors also significantly affected the damping factor of the PNC. Increasing the screw speed while compounding saw an increase in the damping factor however the lowest damping factor of the PNCs was that of the 10 wt.% CNT compounded at 200 rpm, with an increase in the damping factor when compounding at 300 rpm. The lowering of the damping factor indicates good interfacial adhesion between the CNTs and the PC/ABS matrix [26]. At the same time, there was an increase in the damping factor for 10 wt.% CNT at 300 rpm was likely due to the reduction in polymer chain length. While screw speed affected the peak tan delta dampening factor, it did not significantly affect the temperature at which it occurred. However, the peak Tan delta Tg was significantly increased with the increase in CNT concentration, indicating enhanced filler matrix bonding.

The DSC results suggest that the incorporation of CNTs into the PC/ABS matrix causes slight increases in the degree of crystallinity of the PNC. The endothermic peak could be attributed to the melting of crystalline regions of the polymer at 220 °C. The peak intensifies in the second heating, which suggests that the controlled heating and cooling of the sample allows for more crystalline structures to form. The presence of the 002 peak in the PNC XRD plots indicates that a crystalline structure was formed due to the inclusion of CNTs in the PC/ABS matrix. This would suggest that the CNTs are acting as nucleating agents, promoting crystalline structure formation in the polymer matrix. It has been reported in the literature that CNTs show preferential dispersion with PC within the PC/ABS phases [6]. This preferential dispersion likely promotes crystalline structure formation in the PC phase, which can be semi-crystalline. Semi-crystalline PC has a melting of 220–230 °C [28,29,30,31], which correlates with DSC results in the current study, suggesting that the CNTs are promoting crystallinity in the PC phase of the PC/ABS samples. The absence of the 101 peak in the PNC samples suggests that there are no large CNT agglomerates present in the samples, indicating that the CNTs are adequately dispersed in the PC/ABS matrix for all screw speeds. Kasaliwal et al. [4] found that the formation of large agglomerates of CNTs can be reduced at higher mixing speeds and mixing times as the resulting high shear stresses cause the agglomerates to disperse through rupture mechanisms.

As expected, the Charpy impact strength is significantly lower with the incorporation of CNTs into the polymer matrix. This is due to the reduced chain mobility caused by the entangled CNT network, which affects the polymers’ ability to deform while an impact load is applied. With the increased amount of CNTs, agglomerates of CNTs can form, which can lead to brittleness due to defects present in the PNC [6,32]. Screw speed significantly affected the Charpy impact strength of the PNC with the highest impact strength being obtained by compounding at 300 rpm for each CNT concentration. This could indicate that the CNT were undergoing exfoliation/intercalation within the matrix [33]. Compounding at 200 rpm also had the lowest impact strength for each CNT concentration, indicating that higher screw speeds are needed for enhanced dispersion of the CNT. The enhanced exfoliation/intercalation when compounding at higher screw speeds could allow for better polymer chain alignment to occur which may have resulted in higher impact strength.

The introduction of rigid fillers such as CNTs into the PC/ABS matrix increases the PNC’s stiffness. This can be seen with the incremental increase in tensile modulus and the increasing CNT concentration. The tensile modulus of the PNC sees a significant increase with increasing CNT concentration. Dal Lago et al. [6] found that the tensile modulus of the PC/ABS/CNT PNC was enhanced with increasing filler concentration. While screw speed is not a significant factor that affects tensile modulus, the *p*-value nears 0.05 which would suggest that more samples may be required to determine its significance. Similar to the tensile modulus, the tensile strength of the PNC saw an incremental increase with increasing CNT concentration. CNT concentration is a significant factor that affects the tensile strength of the PNC. Screw speed is not a significant factor that affects the tensile strength of the PNC however, the interaction between the two factors is significant. This can be seen in Figure 12, where the tensile strength of the 10 wt.% CNT compounded at 300 rpm sees a significant drop compared to the other compounding screw speeds. This could be attributed to shear degradation occurring at the highest screw speed or excessive chain alignment causing a reduction in ductility. As stiffness increases, with increasing CNT content, a reduction in elongation at break occurs. Increased CNT concentration causes a significant reduction in the elongation at the break of the PNCs. It is widely reported that nanofillers act as nucleating agents, which increase crystallinity and, thereby the elongation at break. With the opposite observed in this work, it is likely that shear degradation of the polymer chain is the predominant factor. This is in agreement with findings from Charpy impact testing, where increasing CNT concentrations, agglomerates of CNTs formed, which increased the brittleness of the PNC [6,32]. Ganß et al. [34] found that strain localisation around the network of CNTs causes a severe modulus mismatch between polymer and filler, reducing the ductile yielding behaviour. The CNTs hinder chain mobility of the PC/ABS matrix and cause a reduction in elongation at break. Screw speed did not significantly affect the elongation at break of the PNCs. This would suggest that the CNTs are adequately dispersed in the PC/ABS matrix for all screw speeds.

CNT concentration also significantly affects the flexural modulus of the PNCs. This behaviour is similar to that of tensile modulus however, unlike tensile modulus, screw speed and the interactions between the two factors significantly affect the flexural modulus. The incorporation of CNTs in the PC/ABS matrix increased the PNCs’ ability to store energy elastically during deformation. This is illustrated through an increase in storage modulus as CNT concentration increases in the PNC. The stiffness of the PNC increases with CNT concentration due to a reduction in chain mobility, which is caused by the entangled CNT networks in the PC/ABS matrix. The flexural modulus of the PNCs increases as screw speed which would suggest that alignment of polymer chains is occurring at higher screw speeds which may also indicate shear degradation as shorter chains may align more readily. This orientation of the polymer chains would create a stiffer PNC, increasing its flexural modulus. While the alignment of the polymer chains increases the stiffness of the PNC, it can also make it brittle, as seen with the elongation at break due to shear degradation. CNT concentration, screw speed and the interaction between these two factors significantly affect the flexural strength. The incorporation of CNTs in the PC/ABS matrix sees an increase in flexural strength; however, at 10 wt.% CNT compounded at 300 rpm, no improvement was observed. This would suggest that the excessive alignment of the polymer chains along with the agglomerates of CNTs at this concentration causes localised strain around the network of CNTs, similar to observations made for elongation at break [34]. This reduction in flexural properties may be attributed to shear degradation at high screw speeds and CNT concentration. The highest flexural strength was observed at 10 wt.% compounded at 200 rpm.

For EMI SE, the only significant factor that affects it is the CNT concentration. The highest level of CNT concentration achieves the best shielding at 2 GHz. Screw speed is not a significant factor that affects the EMI SE of a PNC. There is little to no attenuation occurring with the base polymer; however, it was observed that with the inclusion of CNTs, the attenuation increases. This was due to the CNT concentration being above the percolation threshold range of 0.5 to 4.5 wt.%. Above this range, the CNTs create a conductive network throughout the polymer matrix [7]. With a heterogeneous polymer blend such as PC/ABS, two types of dispersion occur: predominantly in the PC phase and distributed concentrating at the interface of the PC/ABS phases [35,36]. This is known as double percolation, where one polymer phase will have a higher ratio of CNTs, thus increasing the electrical conductivity and the EMI SE of the PNC. Dal Lago et al. [6] showed this preferential dispersion using CNTs and PC/ABS.

As EMI SE is a critical property, 10 wt.% CNT concentration has been selected and as it was evident that the best compounding screw speed for this PNC was 200 rpm as it had the best flexural and tensile strength attributed to the best filler matrix bonding (highest storage modulus) of the PNCs. The 10 wt.% CNT compounded at 200 rpm also had the lowest damping factor of the PNCs, which indicates good interfacial adhesion between the CNT and the PC/ABS matrix [26].

## 5. Conclusions

In conclusion, this study compared the effect of screw speed and CNT concentration on the mechanical, thermal and EMI SE properties of PC/ABS and its PNCs. CNT concentration had a significant influence on the mechanical properties of the PNCs, while extrusion screw speed only significantly affects the Charpy impact strength and the flexural properties of the PNC. The inclusion of CNTs in the polymer matrix lowered the Charpy impact strength while increasing the flexural modulus and strength. As an EMI shielding material, the 10 wt.% PNCs achieved the best result, which is a critical factor for this study, which is to obtain a suitable material for AM EMI shielding constructs. At 10 wt.% CNT compounded at 300 rpm, there was no improvement in flexural strength, which suggests shear degradation of the polymer matrix, or excessive chain alignment caused strain localisation which occurred around the network of CNTs. The thermo-mechanical characterisation of PC/ABS and its PNC were assessed using DMA, and the best interfacial adhesion between matrices and CNTs was obtained at 10 wt.% CNT compounded at 200 rpm. Compounding at 200 rpm also had the best flexural and tensile strength, which was attributed to the best filler matrix bonding (highest storage modulus) of the PNCs. This research contributes valuable insights into the effect of CNT concentration and extrusion screw speed on the mechanical, thermal and EMI SE properties of PC/ABS and its PNCs, through the identification of the competing factors of CNT dispersion and matrix degradation at higher screw speeds.

## Figures and Tables

**Table 1:** Parameter Settings for the design of experiments.

Parameter	Low	Medium	High
Screw Speed (rpm)	100	200	300
CNT Concentration (wt.%)	0	5	10

**Table 2:** DoE run order.

Run Order	Screw Speed (rpm)	CNT Concentration (wt.%)
1	100	0
2	100	5
3	100	10
4	200	0
5	200	5
6	200	10
7	300	0
8	300	5
9	300	10

**Table 3:** The glass transition temperature was obtained using DSC.

Glass Transition Temperature (°C)			
	100 rpm	200 rpm	300 rpm
PC/ABS	111.7	112.8	110.6
5 wt.% CNT	112.6	113.5	112.2
10 wt.% CNT	112.0	110.7	107.8

## Data Availability

Data are contained within the article.
